# Diagnostic challenges in boerhaave syndrome with bilateral pleural contamination: complementary roles of computed tomography, endoscopy, and video-assisted thoracoscopic surgery

**DOI:** 10.3389/fsurg.2026.1917251

**Published:** 2026-08-12

**Authors:** Junxue Han, Ximing Mu, Chaojie Xue, Aofei Yang, Shujuan Bai, Dongde Li

**Affiliations:** School of Medicine, Alar Hospital of Xinjiang Production and Construction Corps, Sir Run Run Shaw Hospital, Zhejiang University, Alar, China

**Keywords:** boerhaave syndrome, computed tomography, endoscopy, esophageal perforation, mediastinitis, pleural effusion, pneumomediastinum, VATS

## Abstract

**Background:**

Boerhaave syndrome is a rare but life-threatening condition due to spontaneous transmural perforation of the esophagus, which most often occurs after copious vomiting. Late diagnosis may lead to mediastinitis, empyema, septicemia, and even death. Localization of the perforation is sometimes difficult despite computed tomography (CT) being regarded as the cornerstone in diagnosis.

**Methods:**

A 36-year-old male Chinese patient presented with acute chest pain following recurrent attacks of violent vomiting due to excessive amount of drinking. On clinical examination, tachypnea, fever, tachycardia, and respiratory distress were observed. Laboratory tests showed significant neutrophilic leukocytosis, raised C-reactive protein and type 2 respiratory failure. Bilateral pleural effusions and pneumomediastinum were seen on contrast-enhanced chest CT, indicative of Boerhaave syndrome. Emergency video-assisted thoracoscopic surgery (VATS), with pleural debridement, drainage, nutritional and intensive care management, was performed. Eventually, an esophageal perforation in the left distal esophageal wall was seen during esophagogastroscopy, revealing the lack of correlation between the distribution of pleural contamination and the site of the perforation. Additional thoracoscopic debridement and multidisciplinary management resulted in progressive recovery.

**Conclusion:**

Boerhaave syndrome is a diagnostic challenge, and radiographic findings do not always reveal the exact site of esophageal perforation. The use of CT, endoscopic evaluation and VATS offered complementary diagnostic and therapeutic benefits, facilitating the proper localization, source control and clinical recovery. Multidisciplinary treatment is key to maximizing the prognosis in the early treatment of this fatal disorder.

## 1 Introduction

Boerhaave syndrome, first reported by the Dutch physician Hermann Boerhaave in 1,724, is a rare but potentially fatal condition in which a perforation occurs in the wall of the esophagus without intervention (1). The syndrome is usually associated with periods of violent vomiting or retching that create a sudden rise in intra-esophageal pressure against a closed cricopharyngeal sphincter. This sudden pressure difference causes a complete perforation of the esophageal wall, usually in the left posterolateral wall of the esophagus at the level of the distal third of the thoracic esophagus. The leakage of gastric and esophageal contents into the mediastinum and pleural cavities results in a rapid onset of inflammatory response that can cause severe mediastinitis, pleural empyema, sepsis and multiorgan dysfunction if not promptly recognized and managed (2, 3).

Boerhaave syndrome is one of the most severe gastrointestinal emergencies, although it is a relatively uncommon type of esophageal injury. The incidence is estimated to be about 3.1 cases per million people per year, but may be underdiagnosed or underreported. Although diagnostic imaging, critical care and surgical procedures have been improved, there is still a high morbidity and mortality associated with the condition. Mortality is more than 40% if treatment is delayed more than 24 h and can be close to 90% in untreated cases, indicating the importance of timely diagnosis and treatment (4, 5).

The clinical findings of Boerhaave syndrome are highly variable and may be nonspecific, thus making the diagnosis difficult. Severe chest or upper abdominal pain is a common complaint after periods of vomiting, overindulgence in food and/or alcohol (2, 6). The classical Mackler's triad of vomiting, lower thoracic pain and subcutaneous emphysema is classically considered pathognomonic, but this is seen in only a minority of patients. Depending on the size and location of the perforation, and the time elapsed between rupture and presentation, additional symptoms may be more prominent, such as dyspnea, tachypnea, fever, pleural effusion, pneumothorax and systemic inflammatory response symptoms. This often results in Boerhaave syndrome having a range of possible mimics, such as acute myocardial infarction, a pulmonary embolism, acute pancreatitis, perforated peptic ulcer, aortic dissection, and spontaneous pneumothorax (7, 8).

Rapid and correct diagnosis depends on clinical suspicion, laboratory tests and imaging studies. Most of the time, the initial modality used is contrast-enhanced computed tomography (CT), which is highly sensitive and can demonstrate the degree of contamination of the mediastinum and pleura. Common computed tomography (CT) findings consist of pneumomediastinum, pleural effusion, esophageal wall thickening, fluid collections within the mediastinum and extraluminal air. Contrast esophagography and upper gastrointestinal endoscopy may be useful to supplement the information obtained to determine the exact location and extent of the perforation. Still, the latter is controversial because it is thought to enlarge the defect (3, 7, 9).

Boerhaave syndrome is a condition that would require a multi-disciplinary approach, including thoracic surgeons, gastroenterologists, radiologists, intensivists and nutrition specialists. Therapy options are conservative therapy, endoscopy and surgery, depending on the time of diagnosis, degree of contamination, patient's stability and experience of the institution. Whatever the modality chosen, the main objectives of the treatment are the rapid control of the contamination, adequate drainage of infected collections, eradication of the sepsis, maintenance of nutritional status and restoration of the integrity of the esophagus (3, 10).

We present here a difficult case of Boerhaave syndrome in a young male patient who was thought to have right-sided pleural contamination, but was found to have a left-sided, distal esophageal perforation. The complexity of the disease when it comes to diagnosis is emphasized, and the complementary use of computed tomography, endoscopic assessment and video-assisted thoracoscopic surgery in reaching a favorable clinical outcome is highlighted.

## 2 Methods

### 2.1 Clinical history and patient information

A 36-year-old male Chinese patient presented to the Emergency Department with acute chest pain of 24 h' duration after a few episodes of forceful vomiting. The symptoms came after heavy drinking. There was no prior history of esophageal disease, gastrointestinal disease, thoracic surgery, chronic medical illness or drug abuse. There was no significant family history. An upper gastrointestinal perforation was on the differential diagnosis list due to the temporal relationship of severe emesis and the onset of chest pain.

### 2.2 First clinical assessment

The patient was acutely ill on admission with signs of respiratory distress. On physical examination, tachypnea and decreased respiratory function were noted. Rough bronchovesicular sounds were auscultated mostly on the right hemithorax. The axillary temperature was 38.3°C, indicative of an ongoing inflammatory process. Vital signs showed a marked physiologic derangement: respiratory rate, 30/min; oxygen saturation, 93% on room air; blood pressure, 154/109 mm Hg; and heart rate, 136 beats/min. This indicated systemic inflammatory response syndrome and warranted a search for a possible thoracic emergency.

History of recent forceful vomiting, severe chest pain, fever and tachycardia with respiratory compromise made it highly suspected as an acute thoracic emergency. The differential diagnosis included acute coronary syndrome, pulmonary embolus, pneumothorax, acute mediastinitis, and esophageal perforation. Since the symptoms and signs were becoming rapidly more severe, and there was evidence of systemic inflammation, the diagnostic work-up was immediately in focus to establish the cause and direct the choice of treatment.

### 2.3 Lab investigations

Laboratory evaluation of the initial laboratory tests indicated marked leukocytosis (64,000) with neutrophilic predominance (88.1%), which is seen in acute infection or severe inflammation. C-reactive protein (CRP) was significantly increased at 108.5 mg/L, which further supported the presence of a significant inflammatory response. Arterial blood gas analysis showed type II respiratory failure with hypercapnia and hypoxemia (PaCO2 56.2 mmHg, PaO2 59.4 mmHg, FiO2 92%). ECG demonstrated sinus rhythm without evidence of acute ischemic changes. Cardiac biomarkers, including cardiac enzymes and troponin, were normal, which excluded acute myocardial infarction as the primary cause of chest pain.

There was marked neutrophilic leukocytosis and raised inflammatory markers with respiratory failure suggestive of a severe inflammatory process, possibly with mediastinal and pleural contamination. Laboratory findings were not specific, but offered important evidence of a potentially life-threatening intrathoracic pathology needing urgent intervention. The inflammatory indices were continuously followed to evaluate treatment response during hospitalization.

### 2.4 Radiologic evaluation and diagnostic considerations

A contrast-enhanced chest computed tomography (CT) was performed as part of the emergency workup. Imaging revealed the presence of heterogeneous bilateral pleural effusions that also included pneumomediastinum. The patient's clinical deterioration and a history of severe vomiting were strongly indicative of esophageal perforation and thus suggested Boerhaave syndrome. The CT examination was also helpful in delineating the extent of the pleural and mediastinal contamination needed for further therapeutic planning.

### 2.5 Multidisciplinary treatment approach

This progressive clinical deterioration led to an emergency consultation by the thoracic surgical team, gastroenterology team, intensive care medicine and anesthesiology. There was a detailed assessment, which indicated that there was an urgent need for surgical management. The right hemithorax was most likely to be contaminated as the right-sided pleural effusion appeared more extensive on CT scans.

The decision to proceed with surgical management was made due to the patient's worsening clinical condition, extensive radiological evidence of pleural contamination and the high risks of severe mediastinitis and the onset of septic complications. In the treatment approach, the multi-disciplinary team emphasized the need for early source control, sufficient drainage of infected collections, respiratory support and early nutritional support.

Then, the patient's right side video-assisted thoracoscopy (VATS) was performed. In operation, large volumes of contaminated pleural content were removed. Four chest tubes were inserted for drainage and irrigation of the thoracic cavity on an ongoing basis. The nasojejunal feeding tube was inserted to allow for sufficient nutrition and to decrease stress on the esophagus. Postoperatively, the patient was transferred to the intensive care unit (ICU) for mechanical ventilatory support and tight hemodynamic surveillance. Immediately, broad-spectrum intravenous antibacterial and antifungal therapy was started.

### 2.6 Clinical course and further interventions

The laboratory parameters significantly improved in the first 10 days following surgery, as the number of neutrophils and CRP reduced. But this positive trend did not continue. The second week post-surgery, inflammation markers went back up, suggesting either ongoing infection or poor source control.

Mortality and disease progression were monitored during the hospital stay with serial clinical assessment, laboratory investigations and radiological examination to evaluate treatment effectiveness. The lack of resolution of inflammatory activity despite initial improvement emphasized the complexity of Boerhaave syndrome and the need for repeated evaluation and prompt change in therapy when there was incomplete source control.

Contrast-enhanced chest CT was repeated, and the amount of pleural fluid in the left thoracic cavity was seen to be increasing. Hence, a second thoracoscopic procedure was carried out to perform further debridement, decortication and irrigation. Esophagogastroscopy revealed that the left distal esophageal wall was ruptured. A three-lumen gastrojejunostomy tube was then placed for enteral feedings, gastric decompression and gastrointestinal secretions.

Recurrent right empyema necessitated further surgical treatment for adequate drainage and infection control, and the patient's hospital course was complicated. An important aspect of management was intensive care management. Other components of ongoing clinical management included the continuous adjustment of respiratory support, fluids, antimicrobial therapy and nutritional supplements, as well as careful surveillance for any postoperative complications, which were dynamic and evolving. This multi-disciplinary approach was a major component of this successful recovery.

### 2.7 Outcome and follow-up

The patient had a gradual improvement with the management provided by a multidisciplinary approach. He spent 25 days in the ICU and was finally transferred to the Thoracic Surgery Department for further recovery. He was discharged in stable condition after another 18 days of inpatient care.

Chest x-ray and gastrointestinal endoscopic examination one month after the operation revealed good recovery and complete healing of the esophageal defect. There was no residual pleural infection, esophageal dysfunction, or significant complications. The patient resumed normal oral intake and daily activities without recurrence, indicating a good clinical outcome after staged multidisciplinary treatment.

## 3 Results

**Figure 1:**
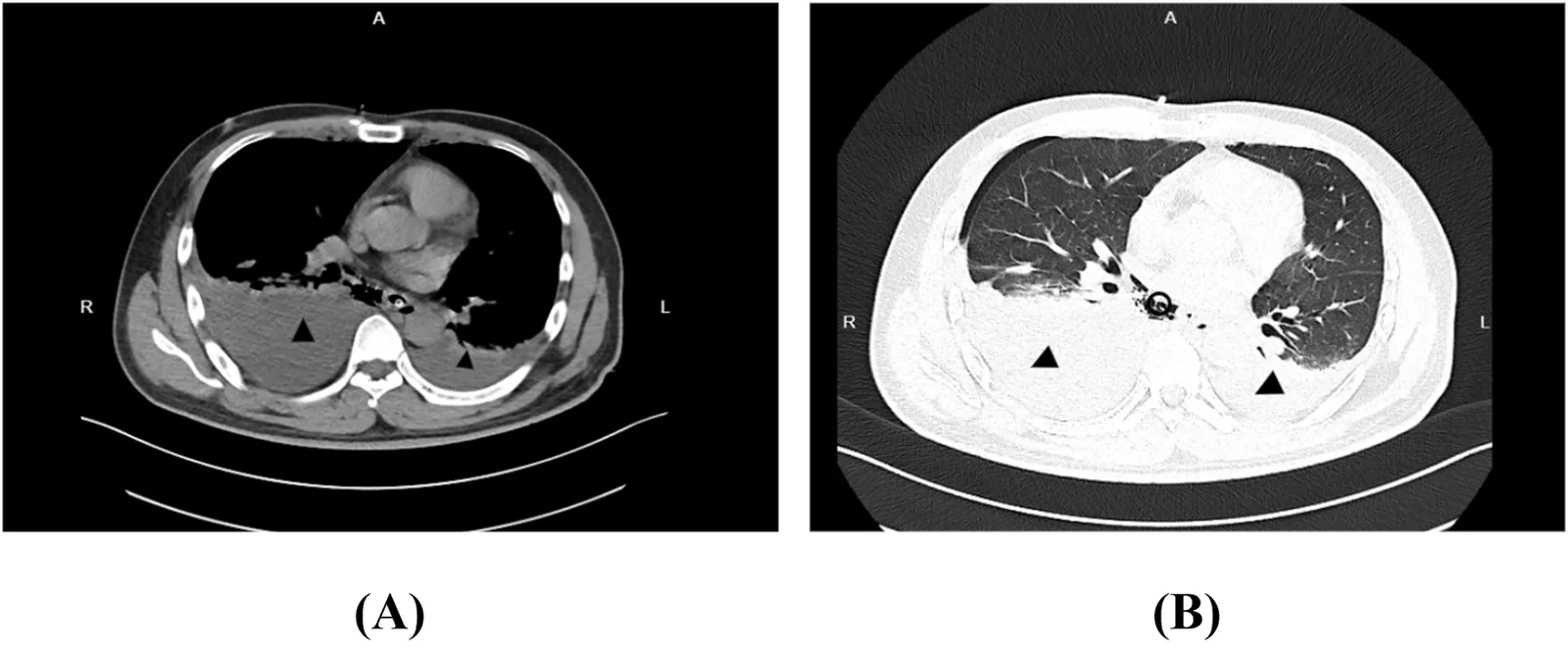
Initial radiological findings in boerhaave syndrome. **(A)** Contrast-enhanced chest CT demonstrating bilateral pleural effusions (▴). **(B)** Lung-window CT image showing bilateral pleural effusions with associated atelectasis (▴) and pneumomediastinum (○).

Initial contrast-enhanced chest CT showed bilateral pleural effusions with associated compressive atelectasis and pneumomediastinum, findings strongly suggestive of esophageal perforation (Figures 1A,B). The perforation was considered to be related to right thoracic involvement based on the extensive pleural contamination observed in the right hemithorax.

The patient initially improved clinically after emergency video-assisted thoracoscopic surgery (VATS), continuous pleural drainage, antimicrobial therapy and intensive care support. In the first 10 days after surgery, neutrophil counts and C-reactive protein levels decreased, indicating a partial control of infection and mediastinal contamination.

**Figure 2:**
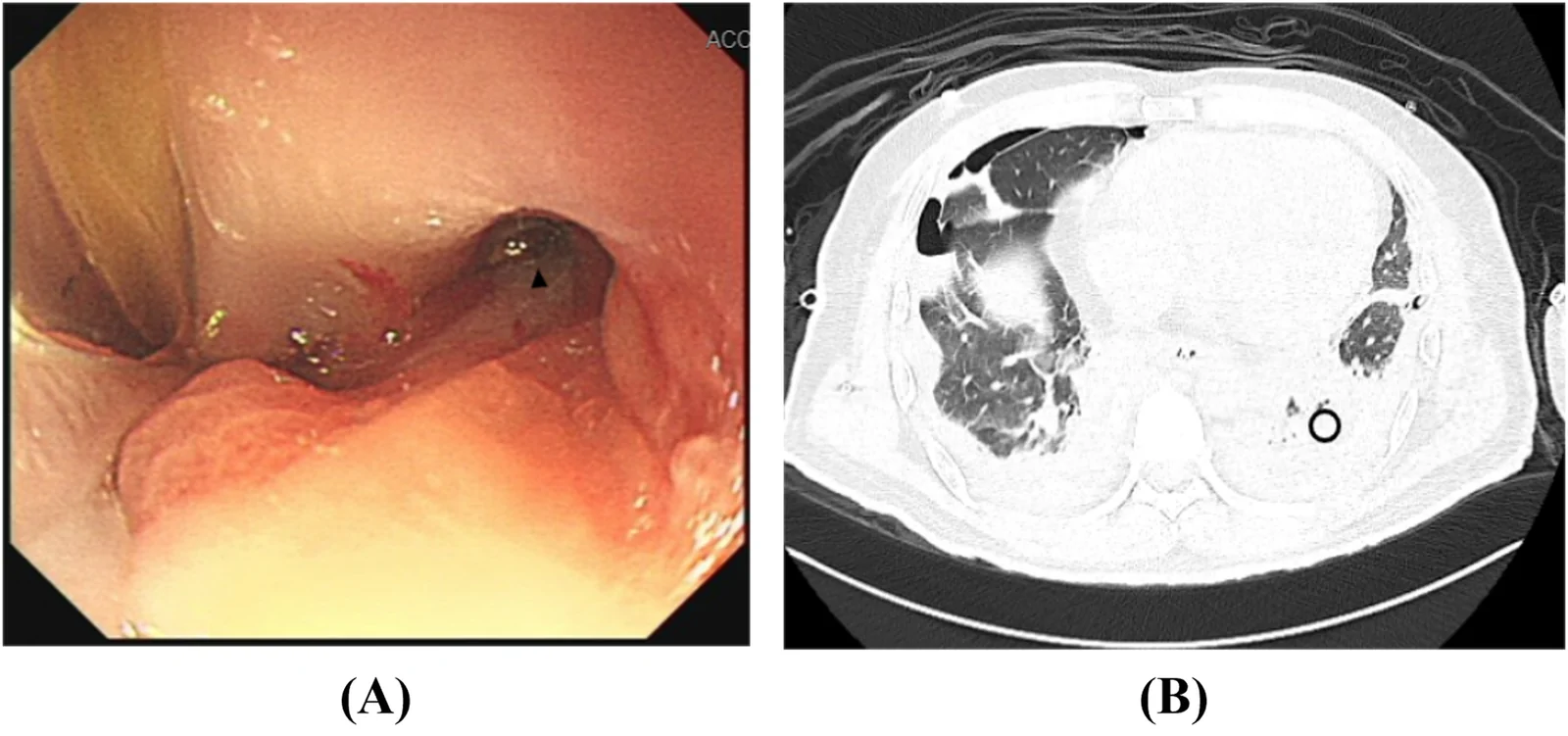
Identification of the esophageal perforation and persistent thoracic contamination. **(A)** Esophagogastroscopy demonstrating a distal esophageal perforation. **(B)** Follow-up chest CT showing persistent left pleural effusion and associated atelectasis (○).

There was an initial response, but inflammatory markers then increased, and the patient deteriorated. Repeat chest CT demonstrated progressive left-sided pleural effusion and persistent atelectatic changes. Subsequent esophagogastroscopy demonstrated a distal esophageal perforation of the left esophageal wall, confirming the source of ongoing contamination (Figures 2A,B).

Further thoracoscopic debridement, drainage procedures, enteral nutritional support and multidisciplinary intensive care management followed a gradual clinical recovery. The patient was admitted for further observation and therapy of recurrent thoracic infection.

**Figure 3:**
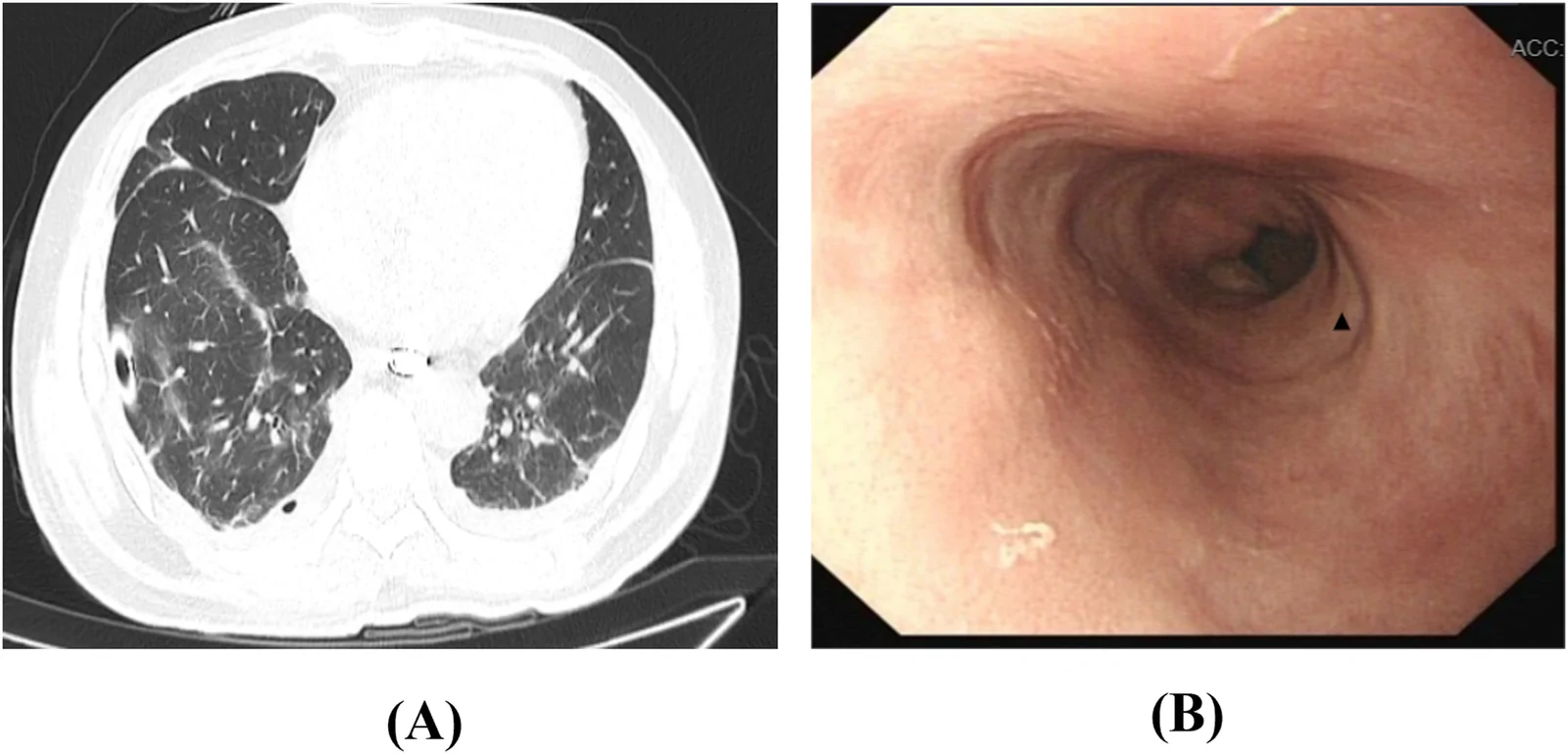
Follow-up evaluation after multidisciplinary treatment. **(A)** Follow-up chest CT demonstrating marked resolution of pleural contamination and satisfactory lung re-expansion. **(B)** Endoscopic image confirming complete healing of the esophageal perforation.

Chest CT at one-month follow-up evaluation revealed marked resolution of pleural pathology and adequate lung re-expansion. Endoscopic examination confirmed complete healing of the esophageal perforation with no evidence of residual leakage (Figures 3A,B). The patient had a good clinical outcome with return to normal oral intake and daily activities without recurrence of symptoms.

## 4 Discussion

Boerhaave syndrome is a rare but potentially devastating condition characterized by spontaneous transmural perforation of the esophagus after a sudden rise in intra-esophageal pressure. Despite significant advances in diagnostic imaging, endoscopic techniques, intensive care management and minimally invasive surgical procedures, the condition still carries significant morbidity and mortality (11–13). The clinical significance of Boerhaave syndrome is not only associated with the severity of the disease itself, but also with the diagnostic difficulties. Delay in diagnosis remains one of the most important predictors of poor outcome, as ongoing contamination of the mediastinum and pleural cavities can rapidly progress to mediastinitis, empyema, septic shock and multiorgan failure (14–16).

The present case emphasizes several important aspects of the diagnosis and management of Boerhaave syndrome. As previously reported, the patient had severe chest pain following episodes of forceful vomiting after drinking excessive alcohol. Alcohol abuse is often cited as a precipitant, as it predisposes to repeated vomiting and sudden increases in intra-esophageal pressure. Sudden pressure gradient against the closed cricopharyngeal sphincter results in full thickness tear of the esophageal wall—the pathophysiological mechanism. The distal posterolateral esophagus is particularly susceptible to rupture, structurally, due to its relative weakness and less support from surrounding tissues (17–19).

One of the most difficult things about Boerhaave syndrome is the variable presentation of the disease. Mackler's triad of vomiting, chest pain and subcutaneous emphysema is classically thought to be pathognomonic, but this triad is present at presentation only in a minority of patients. The first presentation usually is vague and may mimic other thoracic and abdominal emergencies, including acute coronary syndrome, pulmonary embolism, acute pancreatitis, perforated peptic ulcer, spontaneous pneumothorax, or aortic dissection. Thus, clinicians should have a high index of suspicion in patients presenting with acute chest pain following episodes of vigorous vomiting (20–22).

In the present case, laboratory investigations demonstrated marked neutrophilic leukocytosis, elevated inflammatory markers and respiratory failure. These findings, although nonspecific, reflect the severe inflammatory response to mediastinal and pleural contamination. Serial monitoring of inflammatory indices was of clinical value in assessing the response to treatment. The first drop of neutrophil counts and CRP levels was a sign of transient control of the infectious process. At the same time, the later increase was an early marker of persistent contamination and disease progression.

Computed tomography was used a great deal during the patient's clinical course. At present, contrast-enhanced CT is the imaging modality of choice in the assessment of suspected esophageal perforation, as it is able to identify pneumomediastinum, pleural effusions, mediastinal fluid collections, and associated pulmonary complications. Furthermore, CT is useful for assessing the extent of contamination and for planning therapeutic interventions. In our patient, CT findings of bilateral pleural effusions and pneumomediastinum strongly supported the diagnosis of Boerhaave syndrome. But the case also presents an important caveat to imaging studies. CT correctly diagnosed thoracic contamination, but radiological findings initially suggested a more severe involvement of the right pleural cavity and the assumption that the perforation originated from the right side of the esophagus (8, 11, 23, 24).

One of the most instructive things about this case is the discrepancy between the radiological appearance and the true position of the oesophageal defect. Following the first surgical procedure, the patient had a transient clinical improvement and subsequent inflammatory decompensation. Repeat CT imaging subsequently demonstrated progressive left-sided pleural contamination. Endoscopic reassessment revealed a perforation of the left distal esophageal wall. This finding suggests that the distribution of pleural fluid and mediastinal contamination is not necessarily directly related to the anatomical site of the perforation. Therefore, clinicians should be careful in concluding the exact site of rupture based only on imaging findings, especially in patients presenting with bilateral pleural involvement (25, 26).

Another interesting feature of this case is the complementary role of endoscopy and surgery in the diagnosis and management. The role of upper gastrointestinal endoscopy in Boerhaave's syndrome is debated. Some clinicians are concerned that air insufflation may enlarge an existing perforation, but endoscopy allows direct visualization of the esophageal mucosa and accurate localization of the defect. In the present case, endoscopic evaluation was important to identify the exact location of the rupture and to plan the subsequent therapeutic procedures. Importantly, the procedure was performed in the setting of ongoing surgical management, which reduced the risk of delayed intervention (27, 28).

Surgery remains the cornerstone of treatment for patients with extensive contamination, sepsis or late presentation. The main objectives are to eliminate the contamination, sufficiently drain infected collections, prevent further leakage and provide nutritional support. In recent years, video-assisted thoracoscopic surgery has been a useful alternative to conventional thoracotomy. VATS has many benefits when compared to open surgery, such as less surgical trauma, less postoperative pain, shorter recovery time and lower pulmonary complication rates. In the current case, repeated thoracoscopic procedures were successful in debridement and drainage, bypassing the morbidity associated with more invasive surgical approaches (29, 30).

Nutritional support is also an important part of management. When the esophagus is perforated, patients may have to supplement their diet for a while, or for some, there will be a need for other nutritional modalities. When possible, early enteral nutrition is preferred to ensure gastrointestinal tract integrity and prevent infectious diseases and promote wound healing. Adequate nutrition was achieved by placement of a nasojejunal feeding tube followed by a three-lumen gastrojejunal tube, with minimal additional stress on the injured esophagus. The procedures, as well as the wide-spectrum antimicrobial therapy and intensive care support, contributed to the patient's recovery (31, 32).

The multidisciplinary collaboration is another key factor to consider when a successful outcome is achieved in this case. Close collaboration was needed between the thoracic surgeon, gastroenterologist, intensivist, anesthesiologist, radiologist and nursing staff. These group activities enabled prompt decision making, reassessment and adjustment of treatment on an individual basis as the patient's clinical condition changed over time. There is increasing evidence that multidisciplinary care pathways benefit patients with complex thoracic emergencies such as esophageal perforation (26, 33).

## 5 Conclusion

Boerhaave syndrome is a rare but life-threatening entity requiring prompt diagnosis and timely intervention to prevent serious sequelae. This case demonstrates the difficulty in localizing the esophageal perforation by imaging findings only. Computed tomography, endoscopic evaluation, and video-assisted thoracoscopic surgery were used to achieve accurate diagnosis, effective source control, and patient cure. The initial multidisciplinary approach, timely drainage, nutritional support and constant re-evaluation are still crucial for a favorable clinical outcome in patients with Boerhaave syndrome.

## Data Availability

The original contributions presented in the study are included in the article/Supplementary Material, further inquiries can be directed to the corresponding author/s.
